# ECG Holter-based screening for sleep-disordered breathing in post-stroke patients: a large monocentric cohort study

**DOI:** 10.3389/fnins.2026.1973784

**Published:** 2026-10-26

**Authors:** Mathilde Monier, Vincent Pichot, Mathieu Berger, Béatrice Trombert, Pierre Garnier, Jean-Philippe Camdessanché, Frédéric Roche

**Affiliations:** 1 Clinical Physiology and VISAS Center, University Hospital, Saint-Étienne, France; 2 Inserm Sainbiose U1059, Mines, Jean Monnet University, Saint-Étienne, France; 3 Department of Public Health, University Hospital, Saint-Étienne, France; 4 Department of Neurology, University Hospital, Saint-Étienne, France; 5 Inserm CRNL, Fluid Research Team, Jean Monnet University, Saint-Étienne, France

**Keywords:** cardiac monitoring, Holter monitoring, obstructive sleep apnoea, screening, sleep-disordered breathing, stroke

## Abstract

**Introduction:**

Sleep-disordered breathing (SDB) is highly prevalent after stroke and is associated with poor functional outcomes and increased mortality, yet systematic screening remains challenging because of limited polysomnography accessibility. We evaluated three electrocardiogram (ECG)-based indices for SDB screening in a large consecutive post-stroke cohort.

**Methods:**

This retrospective single-centre cohort study included 1,929 consecutive patients hospitalised for stroke or transient ischaemic attack at Saint-Étienne University Hospital (2010–2021) who were in sinus rhythm with ECG signal quality adequate for ECG-derived respiration (EDR) analysis; patients with atrial fibrillation, pacemaker rhythm, or severe ectopy (>10% of beats) were excluded *a priori*. EDR, the Thoracic Impedance Disturbance Index (TIDI) and the Very Low Frequency Increment (VLFI) were compared with the simultaneous oxygen desaturation index (ODI) as reference standard. Sensitivity, specificity, accuracy and predictive values were calculated at ODI thresholds of 15/h and 30/h; a combined TIDI+EDR concordant rule was assessed as a post-hoc exploratory analysis.

**Results:**

The cohort comprised 61% males (mean age 65.9 years). Suspected SDB (ODI ≥ 5/h) was present in 74.6% of patients, moderate-to-severe SDB (ODI ≥ 15/h) in 38.9% and severe SDB (ODI ≥ 30/h) in 17.1%. For ODI ≥ 15/h (*n* = 750), TIDI had the highest sensitivity (93.3%) and negative predictive value (90.8%), whereas EDR had higher specificity (80.2%) and accuracy (74.2%); the combined rule reached 85.2% specificity and 72.9% positive predictive value. For ODI ≥ 30/h (*n* = 330), EDR showed specificity of 92.4% and accuracy of 85.2%, and the combined rule 95.4% and 86.9%, respectively. VLFI performed poorly across all metrics.

**Discussion:**

ECG-based tools can identify post-stroke patients at high risk of SDB using routine cardiac monitoring. The complementary profiles of TIDI (rule-out) and EDR (rule-in) support a sequential screening approach, which requires prospective validation against polysomnography. VLFI is unsuitable for elderly stroke populations because of age-related autonomic dysfunction. Because patients with atrial fibrillation, pacemaker rhythm or severe arrhythmia were excluded, the findings may not generalise to this higher-arrhythmia-burden subgroup, who may also be at higher risk of SDB.

## 1 Introduction

Stroke remains the second leading cause of death and the primary cause of long-term disability worldwide, with approximately 12.2 million new cases annually (GBD 2019 Stroke Collaborators, 2021). In France, approximately 140,000 individuals experience stroke each year, with 20% mortality within 1 year (Béjot et al., 2016). While traditional vascular risk factors (hypertension, diabetes, dyslipidaemia, and smoking) are well established, sleep-disordered breathing (SDB) has emerged as an independent and modifiable risk factor (Yaggi et al., 2005; Redline et al., 2010).

The bidirectional relationship between SDB and stroke is increasingly recognised. Moderate-to-severe obstructive sleep apnoea (OSA) approximately doubles stroke risk (relative risk 2.0–2.2) over 3–10 years (Bassetti et al., 2020). Conversely, SDB prevalence in post-stroke populations reaches 50–70%, substantially exceeding the 23–50% estimated in the general adult population by large polysomnographic cohort studies such as the HypnoLaus study (Seiler et al., 2019; Hasan et al., 2021; Heinzer et al., 2015). This elevated prevalence may reflect both pre-existing undiagnosed SDB and stroke-induced alterations in respiratory control. It should be noted that the meta-analyses cited above (Seiler et al., 2019; Hasan et al., 2021) include studies with highly heterogeneous assessment time points and diagnostic methods, which should be considered when interpreting aggregate prevalence estimates.

Multiple pathophysiological mechanisms link SDB to stroke pathogenesis and poor recovery, including intermittent hypoxaemia, sympathetic activation, endothelial dysfunction, prothrombotic states, and cardiac arrhythmias (Somers et al., 2008; Lévy et al., 2015). Post-stroke SDB is associated with impaired functional recovery and increased mortality (Bassetti et al., 2006; Festic et al., 2018). Continuous positive airway pressure (CPAP) therapy may reduce recurrent vascular events, although randomised trial results remain mixed, largely due to poor adherence (Martínez-García et al., 2005; McEvoy et al., 2016).

Despite this evidence, systematic SDB screening in acute stroke units remains uncommon. Polysomnography, the gold standard, requires specialised equipment limiting routine use (Kapur et al., 2017). In contrast, ECG Holter monitoring is routinely performed in most stroke patients for arrhythmia detection within 24–72 h of admission, creating an opportunity for opportunistic SDB screening.

Three ECG-based algorithms have been proposed. The Thoracic Impedance Disturbance Index (TIDI) quantifies respiratory effort variations, demonstrating 96.8% sensitivity against polysomnography (Poupard et al., 2012). The Very Low Frequency Increment (VLFI) analyses heart rate variability patterns, with 87% sensitivity in middle-aged populations (Roche et al., 2002). ECG-derived respiration (EDR) reconstructs respiratory patterns from QRS morphology changes and has shown accuracy in cardiac populations but has not been systematically evaluated in post-stroke patients (Pichot et al., 2015; Moody et al., 1986).

We aimed to compare the diagnostic performance of EDR, TIDI, and VLFI for SDB screening in a large consecutive post-stroke cohort, using simultaneous oxygen desaturation index (ODI) as the reference standard, and to assess the feasibility of integrated ECG-oximetry screening during routine post-stroke care.

## 2 Materials and methods

### 2.1 Study design and ethical approval

This retrospective, single-centre observational cohort study was conducted at the Neurovascular Unit of Saint-Étienne University Hospital, France. The study received approval from the local ethics committee (Terre d’Éthique, IRBN592022/CHUSTE, 18 May 2022) and the French National Commission on Informatics and Liberty (CNIL, 25 August 2022). All eligible patients received written information by mail with an opt-out mechanism. The study adhered to the Declaration of Helsinki and STROBE guidelines.

### 2.2 Study population

Adult patients (≥18 years) hospitalised for ischaemic stroke, haemorrhagic stroke, or transient ischaemic attack between January 2010 and December 2021 who underwent ECG Holter monitoring with integrated oximetry (VistaO2, Novacor, Rueil-Malmaison, France) during acute hospitalisation were eligible (*n* = 3,595 records retrieved). Because reliable EDR computation requires a predominantly sinus rhythm with stable QRS morphology, patients presenting atrial fibrillation (*n* = 347), a paced rhythm (*n* = 63), or severe ectopy/other arrhythmia exceeding 10% of beats (*n* = 295) during the recording were excluded *a priori* from the entire analytic cohort (*n* = 705 excluded at this step); this exclusion therefore applies uniformly to all three ECG-derived indices (TIDI, EDR, VLFI) and to the ODI reference standard, which were all computed in the same analytic population. A further 141 patients were excluded for missing age or hospitalisation-duration data, and 820 patients were excluded for inadequate ODI/TIDI signal quality (thoracic impedance or SpO2 signal duration <5 h or <70% of total recording duration), yielding the final analytic cohort of 1,929 patients (Figure 1). Because TIDI, EDR, VLFI and ODI were evaluated in the same 1,929 patients, no further per-index denominator differences apply beyond those shown in Figure 1.

**Figure 1:**
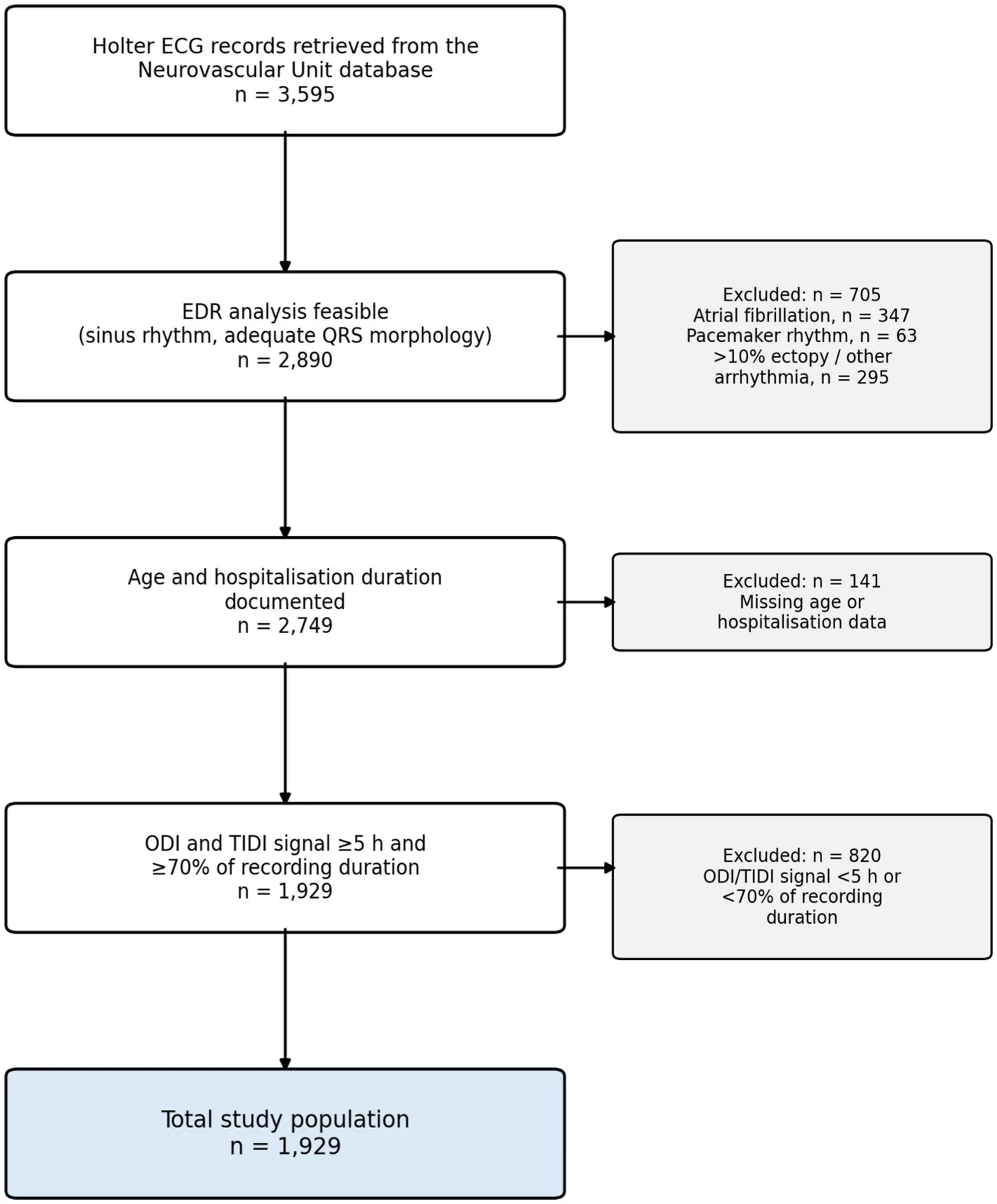
Study flow chart. Of 3,595 Holter ECG records retrieved from the Neurovascular Unit database (January 2010–December 2021), 705 were excluded because atrial fibrillation (*n* = 347), pacemaker rhythm (*n* = 63), or major ectopy/other arrhythmia (*n* = 295) precluded reliable EDR analysis; a further 141 were excluded for missing age or hospitalisation-duration data; and 820 were excluded for inadequate ODI/TIDI signal quality (<5 h or <70% of recording), yielding a final study population of 1,929 patients.

This exclusion means the present findings characterise SDB screening performance specifically in stroke patients without atrial fibrillation, pacemaker rhythm, or major arrhythmia; patients with these rhythm disturbances—who may also be at elevated risk of SDB could not be evaluated with these ECG-derived tools and are discussed as a limitation (see Discussion).

### 2.3 ECG Holter monitoring and oximetry

Continuous ECG monitoring was performed using the VistaO2 device (Novacor), recording 2–3 lead ECG (200 Hz sampling rate), thoracic impedance, and oxygen saturation simultaneously. Chest electrodes were positioned at the fifth right intercostal space mid-axillary line and the first left intercostal space below the clavicle. Oxygen saturation was recorded via a wireless Bluetooth wrist oximeter (Nonin WristOx2 3,150) at 1 Hz. The ECG, thoracic impedance, and SpO2 signals were acquired synchronously on a single time-referenced recording file by the VistaO2 system via Bluetooth, enabling direct temporal alignment of all derived indices without post-hoc synchronisation. Recordings were initiated within 24–72 h of admission and continued for 18–48 h.

### 2.4 Sleep-disordered breathing assessment

#### 2.4.1 Reference standard—oxygen desaturation index (ODI)

ODI was calculated as the number of ≥3% oxygen desaturation events per hour of recording. SDB severity was classified per American Academy of sleep medicine criteria: none (ODI < 5/h), mild (ODI 5–14.9/h), moderate (ODI 15–29.9/h), and severe (ODI ≥ 30/h) (Berry et al., 2012).

#### 2.4.2 Thoracic impedance disturbance index (TIDI)

TIDI quantifies the number of thoracic impedance disturbances per hour. A constant alternating current (1 mA, 50 kHz) measures voltage changes reflecting respiratory effort. The impedance signal underwent cubic spline filtering to remove cardiogenic artefacts. Detection used the Holter device’s validated algorithm (Poupard et al., 2012).

#### 2.4.3 Very low frequency increment (VLFI)

VLFI analyses heart rate variability in the very low frequency spectrum (0.003–0.04 Hz), which increases during apnoeic events due to autonomic oscillations (Guilleminault et al., 1984). A validated threshold of ≥4% identified OSA (Roche et al., 2002). VLFI was computed using HRVanalysis software (Pichot et al., 2016). As detailed in Section 2.2, patients with atrial fibrillation, pacemaker rhythm, or severe ectopy were already excluded from the whole analytic cohort at the inclusion stage; VLFI was therefore computed in the same *n* = 1,929 patients as TIDI and EDR, without a further rhythm-based exclusion specific to this index.

#### 2.4.4 ECG-derived respiration (EDR)

EDR reconstructs respiratory patterns from QRS morphology changes induced by thoracic movements (Moody et al., 1986). Signal processing involved: (1) ECG baseline removal (high-pass filtering) and R-peak detection; (2) calculation of a respiratory surrogate signal from beat-to-beat QRS complex area; (3) automated scoring of events with ≥50% peak-to-peak amplitude reduction lasting ≥10 s or inverted U-shaped patterns (Pichot et al., 2015; Kesper et al., 2012). An apnoea–hypopnoea index (AHI_EDR_) was calculated as events per hour of selected sleep time using custom Python 3.6 functions. Full filter specifications, R-peak detection thresholds and the event-scoring pseudocode are extensively describe in the paper of Pichot et al. (2016).

The nocturnal sleep period was identified manually by a single trained analyst (M. M.) using recording timestamps (typically 23:00–06:00), heart rate patterns, and patient position data where available, in the absence of actigraphy or electroencephalographic staging. Device-derived body position data were not recorded or validated across the whole cohort.

A combined TIDI+EDR classification rule was additionally defined *a priori* as “positive” when both TIDI and EDR were independently positive at the corresponding ODI threshold, and “negative” otherwise; its diagnostic performance was assessed against ODI as an exploratory secondary analysis (see Results, Section 3.4).

### 2.5 Statistical analysis

Descriptive statistics are presented as mean ± standard deviation (SD) for continuous variables and *n* (%) for categorical variables. Comparisons by sex were performed using two-sample *t*-test or Wilcoxon rank sum test for continuous measures, and Pearson chi-square tests or Fisher exact tests for categorical factors. Bland–Altman analysis evaluated agreement between each ECG-derived index and ODI across the whole analytic cohort (n = 1,929), calculating mean bias, 95% limits of agreement, and the corresponding linear regression equation (Bland and Altman, 1986). Diagnostic performance metrics [sensitivity, specificity, accuracy, positive predictive value (PPV), negative predictive value (NPV)] were calculated at ODI thresholds of ≥15/h (moderate-to-severe SDB) and ≥30/h (severe SDB), for TIDI, EDR, VLFI individually and for the combined TIDI+EDR concordant rule. Optimal cut-points were determined using Youden’s index. Analyses were performed using MATLAB R2016b (MathWorks, Natick, MA, USA). Two-tailed *p*-values <0.05 were considered statistically significant.

## 3 Results

### 3.1 Study population and flow

Between 2010 and 2021, 3,595 patients were hospitalised in our Neurovascular Unit and had a Holter recording retrieved. Of these, 705 were excluded because atrial fibrillation (*n* = 347), pacemaker rhythm (*n* = 63), or severe ectopy/other arrhythmia (*n* = 295) during the recording precluded reliable EDR analysis; a further 141 were excluded for missing age or hospitalisation-duration data; and 820 were excluded for inadequate ODI/TIDI signal quality (duration <5 h or <70% of recording), yielding a final analytic cohort of 1,929 patients (Figure 1). The 820 patients excluded at the final step for inadequate signal quality represented 29.8% of the 2,749 patients who reached this stage of screening. The cohort included 1,440 ischaemic strokes, 440 haemorrhagic strokes, and 49 TIAs. The cohort comprised 1,169 males (61%) and 760 females (39%), with a mean age of 65.9 ± 14.5 years (range 18–94). Mean hospitalisation duration was 14.0 ± 13.1 days. Dyslipidaemia was more frequently identified as a stroke risk factor in men, who also presented more frequently with haemorrhagic stroke and less frequently with ischaemic stroke. TIA was a rarely observed stroke subtype, with no difference based on gender. Table 1 presents descriptive statistics for all SDB indices stratified by sex.

**Table 1:** Baseline characteristics and sleep-disordered breathing indices stratified by sex (*n* = 1,929).

Variable	All (*n* = 1,929)	Female (*n* = 760)	Male (*n* = 1,169)
Age (years), mean ± SD	65.9 ± 14.5	66.8 ± 16.2	65.3 ± 13.8
Comorbidities, *n* (%)			
Hypertension	1,397 (72.4)	549 (72.2)	848 (72.5)
Dyslipidaemia	1,294 (67.1)	479 (63.0)	815 (69.7)*
Diabetes	386 (20.0)	152 (20.0)	234 (20.0)
Atrial fibrillation (history)^a^	517 (26.8)	152 (20.0)	365 (31.2)*
Stroke subtype, *n* (%)			
Ischaemic	1,440 (74.7)	577 (75.9)	863 (73.8)*
Haemorrhagic	440 (22.8)	163 (21.5)	277 (23.7)*
TIA	49 (2.5)	20 (2.6)	29 (2.5)
Hospitalisation (days), mean ± SD	14.0 ± 13.1	14.6 ± 12.7	13.6 ± 13.3
ODI (events/h), mean ± SD	15.6 ± 15.3	14.9 ± 15.3	16.0 ± 15.3
TIDI (events/h), mean ± SD	25.7 ± 15.9	23.4 ± 15.6	27.2 ± 16.0
AHI_EDR_ (events/h), mean ± SD	15.8 ± 15.0	13.9 ± 14.1	17.0 ± 15.4
VLFI (%), mean ± SD	3.8 ± 3.5	2.8 ± 2.7	4.5 ± 3.9

AHI_EDR_, apnoea–hypopnoea index derived from ECG-derived respiration; ODI, oxygen desaturation index; SD, standard deviation; TIDI, Thoracic Impedance Disturbance Index; VLFI, Very Low Frequency Increment; TIA, transient ischaemic attack. *p < 0.05 vs female.

a Historical/pre-existing diagnosis, distinct from rhythm during the Holter recording (see Results, Section 3.1); no patient in this final cohort was in atrial fibrillation during the recording itself.

Table 1 “Atrial fibrillation” comorbidity row refers to a pre-existing/historical diagnosis of AF as a vascular risk factor and is distinct from the rhythm observed during the Holter recording itself: patients found to be in AF, paced rhythm, or major arrhythmia during the recording were excluded *a priori* (Section 2.2, Figure 1), so no patient in the final *n* = 1,929 cohort was in atrial fibrillation at the time of ECG-derived index computation. Admission NIHSS and mRS at discharge from the Neurovascular Unit database is unavailable.

### 3.2 Suspected sleep-disordered breathing prevalence

Estimated ODI-based SDB severity distribution in the final cohort (*n* = 1,929): ODI < 5/h in 25.4% (*n* = 490), mild SDB (ODI 5–14.9/h) in 35.7% (*n* = 689), moderate SDB (ODI 15–29.9/h) in 21.8% (*n* = 420), and severe SDB (ODI ≥ 30/h) in 17.1% (*n* = 330). Overall, 74.6% had at least mild suspected SDB (ODI ≥ 5/h), 38.9% had suspected moderate-to-severe SDB (ODI ≥ 15/h, *n* = 750, combining the moderate and severe strata), and 17.1% had suspected severe SDB (ODI ≥ 30/h). The distribution differed modestly by sex: women (*n* = 760) showed 64.5% with ODI < 15/h and 35.5% with ODI ≥ 15/h (16.8% severe), while men (*n* = 1,169) showed 58.9% with ODI < 15/h and 41.1% with ODI ≥ 15/h (17.3% severe) (Table 2).

**Table 2:** ODI-based sleep-disordered breathing severity distribution, overall and by sex (*n* = 1,929).

Group	ODI <5/h	ODI 5–14.9/h	ODI 15–29.9/h	ODI ≥30/h	<15/h total	≥15/h total
All (*n* = 1,929)	490 (25.4%)	689 (35.7%)	420 (21.8%)	330 (17.1%)	61.1%	38.9%
Female (*n* = 760)	209 (27.5%)	281 (37.0%)	142 (18.7%)	128 (16.8%)	64.5%	35.5%
Male (*n* = 1,169)	281 (24.0%)	408 (34.9%)	278 (23.8%)	202 (17.3%)	58.9%	41.1%

ODI, oxygen desaturation index.

### 3.3 Agreement between ECG-derived indices and ODI

Bland–Altman and linear regression analyses were performed across the whole analytic cohort (*n* = 1,929). EDR showed a mean bias of −0.15 events/h (SD 13.29) against ODI, with 95% limits of agreement of approximately −26.3 to +26.0 events/h, and a linear regression of AHI_EDR_ = 0.600 × ODI + 6.389 (*r*^2^ = 0.38, *p* < 0.001) (Figure 2). TIDI showed a mean bias of −10.07 events/h (SD 12.54) against ODI, with 95% limits of agreement of approximately −34.6 to +14.5 events/h, and a linear regression of TIDI = 0.708 × ODI + 14.637 (*r*^2^ = 0.46, *p* < 0.001) (Figure 3), indicating a systematic overestimation of ODI by TIDI at low values and relatively better agreement at higher ODI values. The wide limits of agreement for both indices preclude their use as quantitative AHI estimators; their clinical value lies in categorical rule-out/rule-in performance rather than numerical accuracy (see Section 3.4).

**Figure 2:**
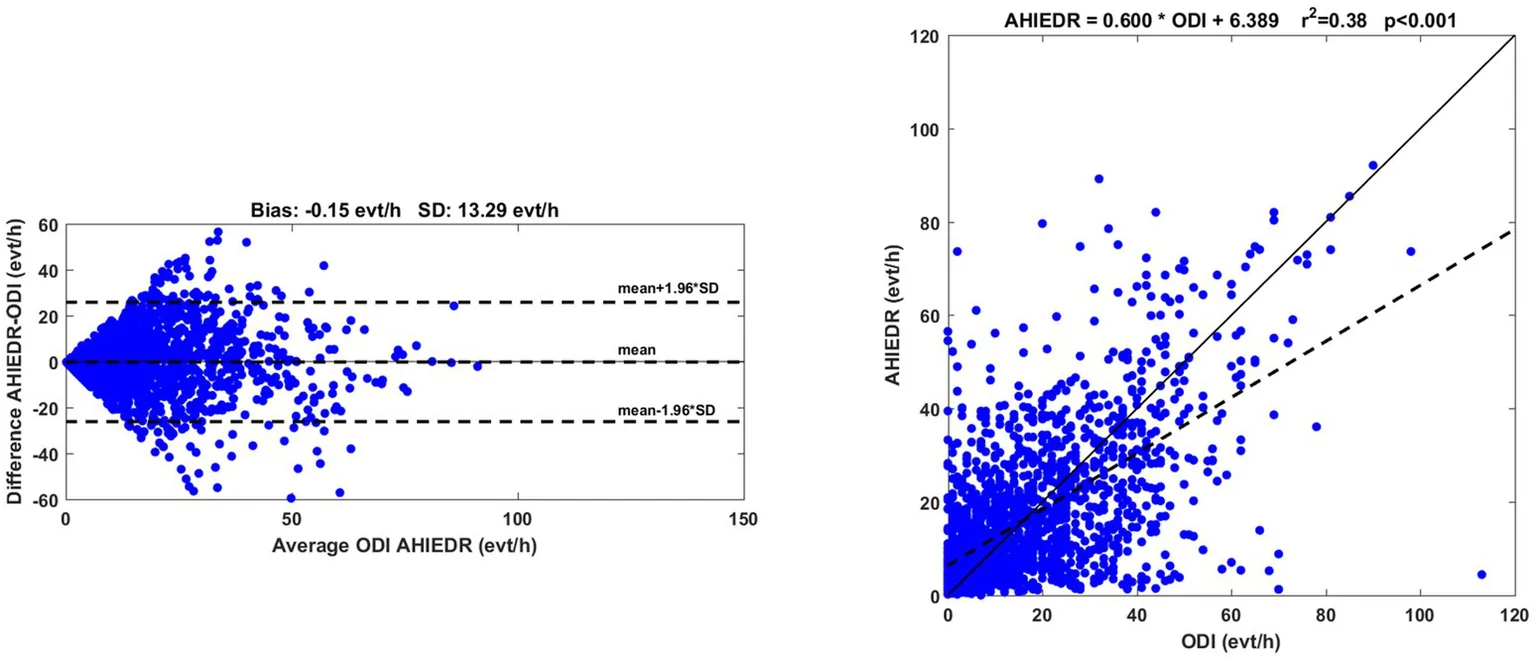
Agreement between AHI_EDR_ and ODI across the whole analytic cohort (*n* = 1,929). Left: Bland–Altman plot (mean bias −0.15 events/h, SD 13.29; dashed lines: 95% limits of agreement). Right: correlation between AHI_EDR_ and ODI (AHI_EDR_ = 0.600 × ODI + 6.389; *r*^2^ = 0.38, *p* < 0.001; solid line: identity; dashed line: regression).

**Figure 3:**
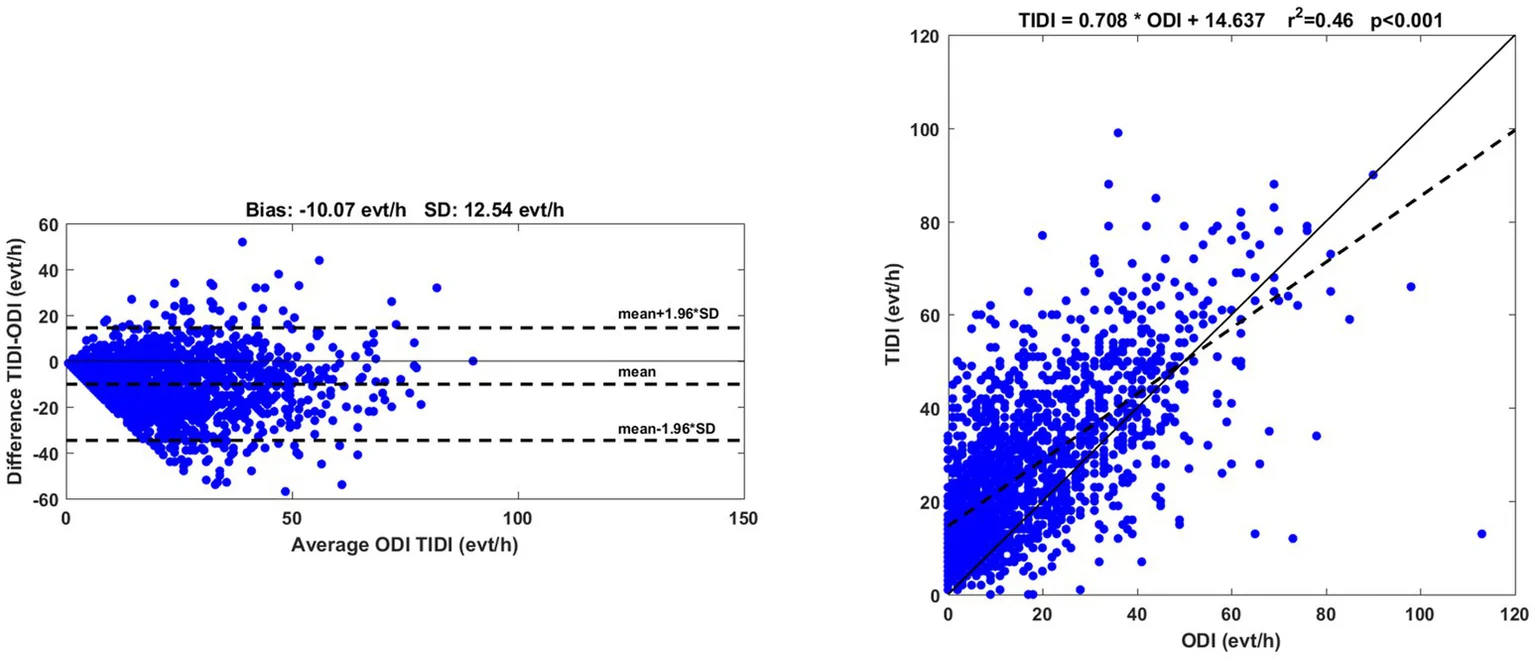
Agreement between TIDI and ODI across the whole analytic cohort (*n* = 1,929). Left: Bland–Altman plot (mean bias −10.07 events/h, SD 12.54; dashed lines: 95% limits of agreement). Right: correlation between TIDI and ODI (TIDI = 0.708 × ODI + 14.637; *r*^2^ = 0.46, *p* < 0.001; solid line: identity; dashed line: regression).

### 3.4 Diagnostic performance

Diagnostic performance metrics are presented in Table 3 [*n* = 750 for ODI ≥ 15/h (moderate + severe combined), *n* = 330 for ODI ≥ 30/h (severe only)]. For moderate-to-severe suspected SDB (ODI ≥ 15/h), TIDI achieved the highest sensitivity (93.3%) and NPV (90.8%), while EDR demonstrated superior specificity (80.2%), accuracy (74.2%), and PPV (67.5%). The combined TIDI+EDR concordant rule reached 85.2% specificity, 76.4% accuracy, and 72.9% PPV, at the cost of lower sensitivity (62.5%) than TIDI alone. For severe suspected SDB (ODI ≥ 30/h), TIDI maintained high sensitivity (83.6%) with excellent NPV (95.8%), while EDR showed outstanding specificity (92.4%) and accuracy (85.2%). The combined rule further improved specificity (95.4%), accuracy (86.9%) and PPV (67.3%) at this threshold, though sensitivity fell to 45.5%. VLFI demonstrated consistently poor performance across all metrics and thresholds (accuracy 52.0–58.0%).

**Table 3:** Diagnostic performance of ECG-derived indices, including the combined TIDI+EDR rule, against oxygen desaturation index at two clinically relevant thresholds.

Index	Sensitivity (%)	Specificity (%)	Accuracy (%)	PPV (%)	NPV (%)
ODI ≥15/h Moderate-to-severe SDB (*n* = 750)					
TIDI	93.3	42.0	61.9	50.6	90.8
EDR	64.9	80.2	74.2	67.5	78.2
VLFI	36.9	61.5	52.0	37.9	60.6
TIDI+EDR (combined)	62.5	85.2	76.4	72.9	78.1
ODI ≥30/h Severe SDB (*n* = 330)					
TIDI	83.6	77.1	78.2	43.0	95.8
EDR	50.0	92.4	85.2	57.7	90.0
VLFI	38.0	62.2	58.0	17.1	83.0
TIDI+EDR (combined)	45.5	95.4	86.9	67.3	89.4

EDR, ECG-derived respiration; NPV, negative predictive value; ODI, oxygen desaturation index; PPV, positive predictive value; TIDI, Thoracic Impedance Disturbance Index; VLFI, Very Low Frequency Increment; SDB, sleep-disordered breathing. The TIDI+EDR combined rule classifies a patient as positive only when both indices are independently positive at the corresponding threshold.

## 4 Discussion

This large monocentric cohort of 1,929 consecutive post-stroke patients free of atrial fibrillation, pacemaker rhythm, or major arrhythmia demonstrates three key findings: (1) the estimated burden of suspected SDB is substantial, affecting 74.6% at any severity and 38.9% with moderate-to-severe disease; (2) ECG-based screening tools show complementary diagnostic characteristics, with TIDI optimised for sensitivity and EDR for specificity, and their combination further improves specificity; and (3) routine cardiac monitoring can be leveraged for opportunistic SDB screening without major additional burden, in patients without significant arrhythmia.

Our observed suspected SDB prevalence is somewhat lower than the pooled estimates from contemporary meta-analyses. Seiler et al. (2019) analysed 89 studies encompassing 7,096 stroke/TIA patients, reporting 71% prevalence for any SDB and 30% for severe SDB; Hasan et al. (2021) confirmed similar estimates across 132 studies with 14,032 patients. Our any-SDB estimate (74.6%) is broadly consistent with these figures, but our moderate-to-severe (38.9%) and severe (17.1%) estimates are somewhat lower than some published pooled estimates. This difference plausibly reflects, at least in part, the *a priori* exclusion of patients with atrial fibrillation, pacemaker rhythm, or major arrhythmia from our analytic cohort (see Limitations): these patients cannot be evaluated with the ECG-derived indices used here, and if they carry a higher burden of severe SDB—as suggested by the pathophysiological overlap between arrhythmia, autonomic dysfunction, and SDB—their exclusion would be expected to lower the observed prevalence in the analysable cohort relative to the true prevalence in the full post-stroke population.

TIDI demonstrated high sensitivity (93.3% for ODI ≥ 15/h; 83.6% for ODI ≥ 30/h) with correspondingly high NPV (90.8 and 95.8%), positioning it as an effective rule-out screening tool. These findings replicate the original TIDI validation by Poupard et al. (2012). The systematic overestimation observed in Bland–Altman analysis likely reflects TIDI’s detection of subclinical respiratory effort disturbances not meeting full hypopnoea criteria by oximetry alone, which is acceptable in a sensitivity-optimised screening context. Damy et al. (2010) have already clearly described, in a screening study conducted on patients with chronic heart failure, the limitations of using TIDI to screen for obstructive or central sleep apnoea.

EDR exhibited superior specificity (80.2% for ODI ≥ 15/h; 92.4% for ODI ≥ 30/h) and accuracy (74.2 and 85.2%), positioning EDR as an effective rule-in screening tool, particularly for severe SDB. The wide limits of agreement in Bland–Altman analysis preclude the use of EDR as a quantitative AHI estimator; its clinical value lies in its categorical classification performance. Our EDR metrics differ from those reported by Pichot et al. (2015) in cardiac populations, with lower sensitivity in our stroke cohort possibly reflecting stroke-specific alterations in respiratory patterns or autonomic control.

VLFI demonstrated poor diagnostic performance (sensitivity 36.9–38.0%; accuracy 52.0–58.0%), contrasting with the original validation by Roche et al. (2002). Several factors may explain this discrepancy. First, VLFI was validated in the 40–60 year age range, whereas our cohort mean age was 65 years; the relationship between VLFI and age in our cohort followed a non-linear, inverted-U pattern (Figure 4), with values rising from early adulthood to a peak in the sixth-to-seventh decade before declining in the oldest patients. This pattern is consistent with progressively impaired autonomic responsiveness to apnoeic events with advancing age, though it also suggests that VLFI’s poor performance in this cohort cannot be attributed to a simple linear age effect and may instead reflect a more complex interaction between age, autonomic reserve, and comorbidity burden. Second, VLFI requires intact autonomic nervous system responsiveness, frequently impaired in stroke patients. These findings indicate VLFI should not be used for SDB screening in elderly stroke cohorts with significant cardiovascular comorbidity.

**Figure 4:**
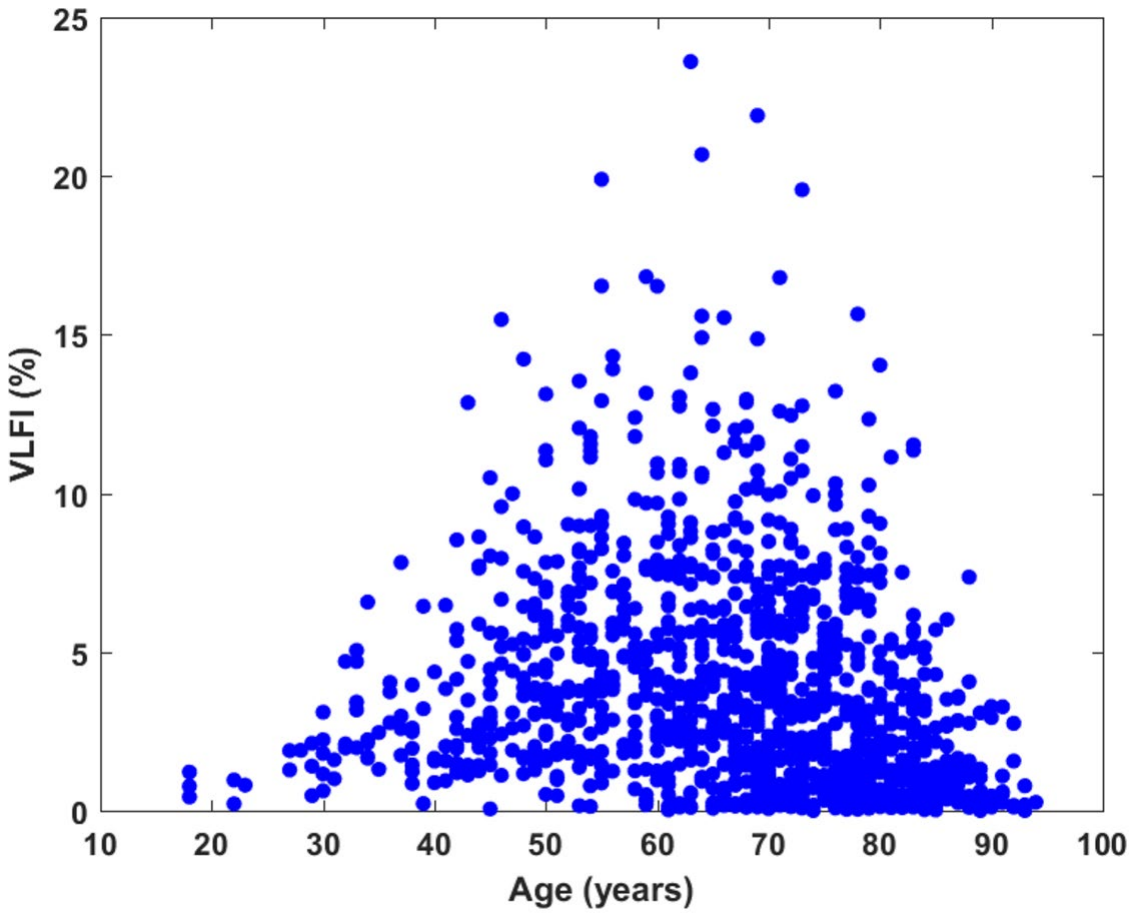
Relationship between age and Very Low Frequency Increment (VLFI). Scatter plot of VLFI (%) against age (years), *n* = 1,929. VLFI values rise from early adulthood to a peak around the sixth-to-seventh decade of life before declining in the oldest patients, describing a non-linear, inverted-U relationship.

Beyond the exclusions already discussed, it should be noted that atrial fibrillation, pacemaker rhythm, and major arrhythmia were not merely a limiting factor for VLFI in this study, but precluded inclusion in the analytic cohort altogether, since reliable EDR computation requires a predominantly sinus rhythm. This is discussed further in the Limitations.

The complementary characteristics of TIDI and EDR suggest a practical sequential screening concept: (1) initial TIDI screening as a high-sensitivity rule-out; (2) EDR confirmation in TIDI-positive cases. We assessed this concept directly as a combined TIDI+EDR concordant classification rule (both positive vs. otherwise): this rule improved specificity and accuracy over either index alone (85.2% specificity / 76.4% accuracy at ODI ≥ 15/h; 95.4% specificity / 86.9% accuracy at ODI ≥ 30/h) at the expense of sensitivity (62.5 and 45.5%, respectively). This supports the rule-in value of concordant TIDI+EDR positivity—for example, to prioritise patients for confirmatory testing or, in centres where validated, for direct auto-CPAP titration—while underscoring that a substantial proportion of true positives would be missed by requiring concordance, so the rule should not be used alone to rule out SDB. In cases of discordance, formal polysomnography or type 2 polygraphy would be warranted to clarify diagnosis before treatment initiation. This combined-rule analysis was exploratory and post-hoc; prospective validation of the sequential algorithm, ideally against polysomnography, remains necessary before clinical implementation.

As already mentioned, sleep apnoea is highly prevalent after acute stroke, and growing evidence suggests that its early identification may meaningfully influence functional recovery. Parra et al. (2011) conducted a randomised controlled trial showing that early treatment of OSA was associated with favourable neurological and functional outcomes, supporting the rationale for early intervention rather than delayed management. Ryan et al. (2011) extended these findings by demonstrating that CPAP use during inpatient rehabilitation improved functional recovery in stroke patients with comorbid OSA, underscoring the importance of not postponing treatment until the chronic phase. Similarly, Bravata et al. (2011) evaluated CPAP as a novel therapeutic approach specifically in the acute ischaemic stroke setting, reporting encouraging feasibility and outcome data that further support early screening and treatment.

Beyond therapeutic considerations, practical screening tools have also been developed to facilitate early detection. Chouchou et al. (2013) showed that thoracic impedance combined with oximetry within a multimodal ECG Holter system can reliably detect respiratory events, offering a minimally invasive option validated in a population with daily symptoms highly predictive of moderate to severe OSAS. This is consistent with the work of Iranzo et al. (2002), who demonstrated that sleep apnoea is already frequent and clinically relevant as early as the first night following cerebral infarction, reinforcing the case for systematic screening from the acute phase onward.

Taken together, these findings argue strongly in favour of early, rather than delayed, screening and management of sleep apnoea after stroke, since untreated OSA may exacerbate cerebrovascular vulnerability and impair rehabilitation potential. However, as highlighted in the recent review by Baillieul et al. (2022), important uncertainties remain regarding optimal timing, patient selection, and long-term treatment adherence, calling for further prospective studies to clarify the balance between early intervention and pragmatic feasibility.

### 4.1 Limitations

Several limitations warrant consideration. ODI served as the reference standard rather than polysomnography-determined AHI; whilst ODI correlates highly with AHI and captures the physiologically most relevant SDB consequence (intermittent hypoxaemia), it may underestimate central apnoeas. As detailed below, the distinction between central and obstructive events cannot be made from ODI alone, and central apnoeas—prevalent in the acute post-stroke period—may have influenced the ODI distributions observed.

Relying on ODI as reference standard carries a further, distinct limitation beyond the central/obstructive distinction discussed below: pulse oximetry itself is susceptible to motion artefact and reduced accuracy during peripheral hypoperfusion, both plausible in acute stroke patients with impaired mobility or vasomotor instability, and this may have introduced additional misclassification into the ODI-based reference standard independent of central-versus-obstructive event type.

A major limitation, clarified in this revision, is that patients with atrial fibrillation, pacemaker rhythm, or major arrhythmia (>10% ectopy) during the Holter recording were excluded from the entire analytic cohort *a priori* (*n* = 705 of the initial 3,595 records, 19.6%), because reliable EDR computation requires a predominantly sinus rhythm with stable QRS morphology. This exclusion applied upstream to the whole cohort and therefore to TIDI, EDR, ODI and VLFI alike. This exclusion has two important consequences. First, it precludes an atrial-fibrillation-versus-no-atrial-fibrillation subgroup comparison of TIDI and EDR performance, since no patient in the final cohort was in atrial fibrillation during the recording. Second, and more importantly, patients with atrial fibrillation, pacemaker dependency, or major arrhythmia are plausibly among those at highest risk of SDB, given the well-described pathophysiological overlap between arrhythmia, autonomic dysfunction, and sleep apnoea; their systematic exclusion may therefore have led to an underestimation of true SDB prevalence in the source population and limits the generalisability of our diagnostic performance estimates to post-stroke patients without major arrhythmia. Evaluating SDB screening tools that do not depend on regular QRS morphology (e.g., oximetry-based or thoracic-impedance-only approaches) would be necessary to extend screening to this excluded, and likely higher-risk, subgroup.

The retrospective design precluded detailed clinical characterisation. Stroke subtypes (ischaemic, haemorrhagic, TIA) are reported in Table 1, but detailed neurological severity scores (NIHSS) and functional outcomes (mRS) were not available from the retrospective database.

The 141 patients excluded due to missing age or hospitalisation-duration data could not be characterised clinically, as the exclusion was driven by data-linkage constraints in retrospective extraction rather than clinical phenotype; selection bias from this specific exclusion is therefore considered unlikely, though it compounds with the arrhythmia-related and signal-quality exclusions discussed above and below. The 820 patients excluded due to inadequate signal quality represent 29.8% of the patients who reached this stage of screening; they may include more severely affected patients who tolerated monitoring less well, and generalisation to the most neurologically impaired patients should be made with caution. The exact time from stroke onset to Holter monitoring initiation was not systematically recorded. Monitoring was performed within 72 h of admission, but interindividual variability in the delay between ictus and hospital arrival means the actual post-stroke timing varied; this is clinically relevant given evidence that SDB severity may evolve in the early post-stroke period.

Sleep period identification for EDR analysis was performed manually by a single trained analyst using recording timestamps and indirect physiological markers, without actigraphy or electroencephalographic staging, precluding assessment of inter-rater reliability. Continuous, validated body-position data were not available. This absence of a continuous posture trace precludes characterisation of positional respiratory events and prevents assessment of the extent to which postural changes may have contributed to false-positive TIDI findings or to morphological artefacts in the EDR waveform.

Finally, our single-centre French cohort may not fully represent international stroke populations; multi-centre validation is warranted.

An important further limitation of our study is the inability to distinguish obstructive from central sleep apnoeas using ODI alone. Central apnoeas, including Cheyne–Stokes respiration, are particularly prevalent in the acute post-stroke period and may constitute up to 25–30% of apnoeic events in some series (Baillieul et al., 2022). Central events have a distinct pathophysiology, a different natural history (many resolve spontaneously during stroke recovery), and different treatment implications. Neither ODI nor the ECG-derived indices evaluated in this study can reliably differentiate central from obstructive events. The term “sleep-disordered breathing” (SDB) has therefore been used throughout this manuscript in preference to “OSA,” to reflect this diagnostic uncertainty. Future studies should use full polysomnography or, at minimum, type 3 polygraphy to phenotype respiratory event types and assess how their relative proportions influence the performance of ECG-based screening indices in the post-stroke population.

## 5 Conclusion

In this large cohort of 1,929 post-stroke patients without atrial fibrillation, pacemaker rhythm, or major arrhythmia, suspected sleep-disordered breathing was prevalent (74.6% at any severity, 38.9% moderate-to-severe) but remains systematically underdiagnosed. ECG-based screening demonstrates complementary utility: TIDI provides high sensitivity (93.3%) and negative predictive value (90.8%) for moderate-to-severe suspected SDB (rule-out tool), whilst EDR offers outstanding specificity (92.4%) and accuracy (85.2%) for severe suspected SDB (rule-in screening tool). A combined TIDI+EDR concordant rule further improves specificity and accuracy relative to either index alone, at the cost of sensitivity, and is proposed as an exploratory, post-hoc rule-in strategy pending prospective validation. TIDI and EDR are derived from the ECG Holter recording and thoracic impedance signal already embedded in the monitoring device used routinely in our stroke unit; the simultaneous acquisition of SpO2 for ODI requires a wrist pulse oximeter, representing a modest but important addition to standard practice. VLFI is not suitable for this elderly comorbid population. As none of the evaluated tools can distinguish obstructive from central apnoeas, and as they could only be evaluated in patients without major arrhythmia—who may not be representative of the highest-risk post-stroke patients—their role is that of individual SDB screening instruments in a defined subgroup, rather than universally applicable diagnostic tools. Implementation of integrated ECG-based SDB screening in stroke units without significant arrhythmia could substantially improve detection rates, enable risk stratification, and potentially enhance functional recovery. Prospective validation against polysomnography, extension of screening approaches to patients with arrhythmia, and clinical outcome studies are needed.

## Data Availability

The dataset for this study can only be made available upon a justified request for reanalysis. The University Hospital of Saint-Étienne, the study sponsor, owns and retains ownership of the data. Requests to access these datasets should be directed to MM, mathilde.monier@chu-st-etienne.fr.
